# A case-control study of the association between the gut microbiota and colorectal cancer: exploring the roles of diet, stress, and race

**DOI:** 10.21203/rs.3.rs-2475944/v1

**Published:** 2023-01-19

**Authors:** Tiffany L Carson, Doratha A Byrd, Kristen S Smith, Daniel Carter, Michael Abaskaron, Rebecca B Little, Sh'Nese Townsend Holmes, William J van Der Pol, Elliot J Lefkowitz, Casey D Morrow, Andrew D Fruge, Maria Gomez

**Affiliations:** Moffitt Cancer Center; Moffitt Cancer Center; Moffitt Cancer Center; Auburn University; Moffitt Cancer Center; University of Alabama at Birmingham; University of Alabama at Birmingham; University of Alabama at Birmingham; University of Alabama at Birmingham; University of Alabama at Birmingham; Auburn University; Moffitt Cancer Center

**Keywords:** colorectal cancer, gut microbiota, disparities, perceived stress, diet, case-control

## Abstract

**Background:**

The gut microbiota is associated with risk for colorectal cancer (CRC), a chronic disease for which racial disparities persist with Black Americans having a higher risk of CRC incidence and mortality compared to other groups. Given documented racial differences, the gut microbiota may offer some insight into previously unexplained racial disparities in CRC incidence and mortality. A case-control analysis comparing 11 women newly diagnosed with CRC with 22 cancer-free women matched on age, BMI, and race in a 1:2 ratio was conducted. Information about participants’ diet and perceived stress levels were obtained via 24-hour Dietary Recall and Perceived Stress Scale-10 survey, respectively. Participants provided stool samples from which microbial genomic DNA was extracted to reveal the abundance of 26 genera chosen *a priori* based on their previously observed relevance to CRC, anxiety symptoms, and diet.

**Results:**

Significantly lower alpha diversity was observed among cancer-free Black women compared to all other race-cancer status combinations. No group differences were observed when comparing beta diversity. Non-Hispanic White CRC cases tended to have higher relative abundance of *Fusobacteria, Gemellaceae,* and *Peptostreptococcus* compared to all other race-cancer combination groups. Perceived stress was inversely associated with alpha diversity and was associated with additional genera.

**Conclusions:**

Our findings suggest that microbiome-CRC associations may differ by racial group. Additional large, racially diverse population-based studies are needed to determine if previously identified associations between characteristics of the gut microbiome and CRC are generalizable to Black women and other racial, ethnic, and gender groups.

## Introduction

Colorectal cancer (CRC) is the 3rd most common cancer among adults living in the United States with approximately 150,000 new cases and more than 50,000 CRC-related deaths annually.^1^ While improvements in screening and treatment have led to a decline in overall CRC incidence and mortality over time, racial disparities persist. For example, the incidence of CRC is approximately 10–30% higher among non-Hispanic Black individuals compared to other racial and ethnic groups.^2,3^ Black individuals also have a lower 5-year overall survival rate after CRC surgery compared to White individuals.^2^ Known risk factors for CRC include increasing age, male sex, family history, inflammatory bowel disease, type 2 diabetes, alcohol consumption, smoking, physical inactivity, high consumption of red and processed meats, high fat diet, and obesity.^3,4^

In recent years, associations between the gut microbiota and CRC have also increasingly been investigated in the etiology and pathology of this chronic disease.^5-7^ The gut microbiota refers to the microbial communities that inhabit the gut and plays a role in digestion, metabolism, nutrient absorption, and immune health. Associations have been identified between the gut microbiota and many chronic diseases including CRC.^5^ Inflammation in the gut can lead to dysplasia which may lead to many chronic diseases including CRC.^8^ When dysplasia occurs, structural damage begins in the epithelium barriers between the microbiota and the immune cells in the lamina propria. This facilitates bacterial translocation which increases exposure of immunogenic microbial compounds to both epithelial cells and antigen-presenting cells.^9^ Therefore, bacteria stimulate immune signaling pathways resulting in a loss of homeostasis and an environment that is prone to neoplasms.

The composition of the gut microbiota is largely influenced at birth by mode of delivery.^10,11^ Additional environmental and behavioral factors are associated with the gut microbiota over the life course including diet, physical activity, medication usage, and stress.^12-15^ Racial and ethnic differences in the gut microbiota are also documented.^13,16,17^ One study comparing the gut microbiota of a generally healthy, racially diverse sample of women reported a higher abundance of Bacteroidetes among Black women compared to White.^13^ This is of importance given the evidence that Bacteroidetes may be enriched among individuals with CRC.^18,19^ Reasons for racial differences are believed to be largely a result of social, environmental, and cultural factors associated with race and ethnicity (e.g., diet, stress).^20,21^ Given the association between the gut microbiota and several chronic diseases for which racial disparities persist, such as CRC, further investigations into the gut microbiota as a potential contributor to health disparities are warranted.

While many risk factors for CRC are known, racial disparities in CRC persist among women even after controlling for these traditional risk factors.^3^ With the mounting evidence supporting an association between the gut microbiota and CRC, the gut microbiota must be integrated into CRC research. Dietary intake, a behavior that is associated with the gut microbiota and tends to differ by race,^22,23^ is also associated with risk for CRC.^24^ Thus, it is plausible that if race is associated with diet which in turn affects the composition of the gut microbiota which is associated with CRC risk, then racial disparities in CRC may be driven by the intersection of diet and the gut microbiota. Similarly, there is increasing evidence to support a relationship between psychological stress, which also tends to differ by race, and the gut microbiota.^13,25^ These links may suggest that the interaction between psychological stress and the gut microbiota may contribute to unexplained racial disparities in CRC.

The purpose of this study was to compare the gut microbiota of a racially diverse sample of women with newly diagnosed CRC to matched cancer-free controls. We compared alpha diversity and other *a priori*-selected microbiota characteristics across a racially diverse sample of women newly diagnosed with CRC and cancer-free controls. We also assessed racial differences in CRC-associated microbiota characteristics, hypothesizing that CRC-associated gut microbiota characteristics would be more prevalent among Black women compared to White women. Lastly, we compared select dietary factors by race-CRC status and examined associations between perceived stress and relative abundances of *a priori*-selected genera.

## Methods

From 2016–2019, we enrolled 11 cis-gendered women with newly diagnosed CRC prior to their initiation of treatment (case) and 178 cis-gendered women from the same geographic area who were cancer free (control). Cases were recruited collaboratively with the multidisciplinary cancer treatment clinic at the University of Alabama at Birmingham. Research staff and clinic staff worked together to identify potential participants with upcoming gastrointestinal oncology confirmatory appointments. After completing the clinic appointment, research staff met with eligible patients to inform them of the study opportunity and invited them to participate. Interested patients were then enrolled in the study and completed the first study visit the same day to limit participant burden. Controls were cancer-free volunteers from the local area that would be served by the same hospital as cases. Controls were recruited using flyers, word of mouth, and small media. Participants self-identified as non-Hispanic Black or White. Exclusion criteria were the following: (1) current tobacco use, (2) current pregnancy, (3) previous cancer diagnosis, or (4) use of antibiotics or other medications known to alter the gut in the previous 90 days. All study-related protocols and questionnaires received approval from The University of Alabama at Birmingham Institutional Review Board for human subjects. Participants provided written informed consent and were compensated US $50 for their time.

For this case-control analysis, CRC patients were matched to cancer-free controls using age, BMI, and race. Cases were matched 1:2 with controls leading to a sample of 33 participants. All participants completed two study visits during which demographics, anthropometrics, survey data, and biospecimens were collected. During visit 1, participants completed the following:

### Demographics survey –

Age; race and ethnicity; education (less than high school, high school/general equivalency diploma, some college, Associates, Bachelors, Masters, Doctoral); household income (US <$10,000, $10,000–$19,999, $20,000–$29,999, $30,000–$39,999, $40,000–$49,999, or ≥$50,000); and number of individuals in the household were collected using a standardized demographics data collection survey.

### Anthropometric measures –

Weight and height were measured in light, indoor clothing, without shoes, using a calibrated digital measuring station (Seca 284 measuring station, Hanover, MD). Body mass index (BMI) was calculated as weight (kilogram)/height (square meter). Waist circumference was measured to the nearest 0.10 centimeter using the Gulick II tension spring measuring tape (model 67020).

### Perceived Stress Scale-10 (PSS-10) –

Participants’ global perception of perceived stress in the previous month was assessed using the PSS-10, a validated 10-item scale used to assess the degree to which situations in one's life are appraised as stressful.^26^ Participants provided a response (0 = never, 1 = almost never, 2 = sometimes, 3 = fairly often, and 4 = very often) to a series of 10 statements about the occurrence of stressful events. Higher scores on the PSS-10 indicate greater perceived stress (possible range 0–40). The PSS has good internal consistency (Cronbach's α = 0.86) and is positively correlated with other indices of stress among adults.^27^

At the end of the first study visit, participants were given a stool collection kit along with verbal and written instructions for proper sample collection at home. Participants were asked to collect one fecal sample at home and return at the next study visit which was scheduled within the following 5 to 7 days. During the second visit, participants returned stool samples and provided dietary information using the National Cancer Institute Automated Self-administered 24-hour Dietary Recall, which includes multilevel food probes and cues to assess food types and amounts.^28^ Total calories, macronutrient, sugar, and fiber intakes were retrieved from ASA-24 for analyses.

#### Sample Collection

Participants were asked to self-collect stool samples using ParaPak vials (Meridian Biosciences, Inc; Cincinnati, OH) no more than 48 hours before their second clinic visit. Participants were advised to store vials with collected samples in a biohazard bag provided by the study in their home freezer until samples were returned to research staff at the second study visit. Once received by research staff, each sample was diluted to 0.1 mg/ml in Cary-Blair medium for total volume of 20 mL with 10% glycerol by volume. Samples were aliquoted into cryovials and stored at −80°C until time for DNA extraction and processing.

#### DNA Extraction and Illumina MiSeq DNA Sequencing

Microbial genomic DNA was isolated using the Fecal DNA isolation kit from Zymo Research following the manufacturer's instructions. Once the sample DNA was prepared, polymerase chain reaction was used with unique barcoded primers to amplify the variable region 4 (V4) region of the 16S rDNA gene to create an amplicon library from individual samples. The polymerase chain reaction product was approximately 255 bases from the V4 segment of the 16S rDNA gene, and we sequenced 251 bases single-end reads using Illumina MiSeq.

#### Bioinformatics

FASTQ conversion of the raw data files was performed following demultiplexing using MiSeq reporter. Quality control of sequence reads was performed with DADA2 ^29^ and low-quality data filtered out using the function fastqPairedFilter (truncLen = c(240,240), maxN = 0, maxEE = c(2,2), truncQ = 2). Filtering, denoising, and clustering of reads into Amplicon Sequence Variants (ASVs) was done using DADA2. Taxon assignment was performed with Mothur^30^ and the SILVA 16S rDNA database (SILVA_132_QIIME_release).^31-33^ These tools were incorporated into an update of our automated analysis pipeline, QWRAP.^34^ Alpha (Shannon, Simpson, and observed species) and beta diversity (Bray Curtis and weighted and unweighted Unifrac) were calculated using QIIME.^35^

#### Statistical Methods:

We chose 26 genera *a priori* based on their previously observed relevance to CRC, anxiety symptoms, and diet and are listed by phylum as follows: Euryarchaeota - *Methanobrevibacter;* Actinobacteria - *Bifidobacterium;* Bacteroidetes - *Bacteroides, Porphyromonas,* Prevotella; Clostridia - Parvimonas, Peptostreptococcus; Firmicutes- *Eubacterium, [Ruminococcus], Blautia, Clostridium, Coprococcus, Lachnospira, Roseburia, Butyricicoccus, Faecalibacterium, Ruminococcus, [Eubacterium], Clostridium,* Dialister, Gemella, Lactobacillus; Fusobacteria - *Fusobacterium;* Proteobacteria -*Sutterella, Succinivibrio;* Verrucomicrobia - *Akkermansia*.

Statistical analyses were conducted using IBM SPSS Statistics for Windows, Version 25.0. (IBM Corp., Armonk, NY), R version (1.3.1), and figures were generated using GraphPad Prism version 8.0.0 (GraphPad Software, San Diego, CA). Complete data were available for matching from four non-Hispanic Black and seven non-Hispanic White women with CRC and 92 non-Hispanic Black and 86 non-Hispanic White cancer-free women. Controls were frequency-matched 2:1 to cases using the FUZZY extension in SPSS with the following parameters: race- exact; age- +/− 5 years; BMI- +/− 5kg/m^2^.

We conducted descriptive analyses among each of the four race-cancer status combinations for participant characteristics, diet variables, perceived stress, alpha diversity metrics, and relative abundance of genera. Normality was determined using the Shapiro-Wilk test. Overall differences in normally distributed variables were assessed using one-way analysis of variance (ANOVA). Between-group differences for variables with non-normal distribution (genera and food groups) were analyzed using Kruskal-Wallis ANOVA. We used unconditional multivariable logistic regression to estimate the associations of the microbiome metrics with CRC. We included age, race, and body mass index (BMI) in the regression models based on previous literature and biological plausibility. Post-hoc exploratory analyses between stress variables of interest and genera were conducted using partial Spearman correlations. We accounted for multiple testing in all analyses using Bonferroni correction.

## Results

Twenty-two cancer-free women (controls) were race, age, and BMI matched to eleven women who were treatment-naïve CRC patients (cases). As shown in Table 1, there were no significant differences in age or BMI when comparing cases and controls, which was expected due to our matching criteria. Waist circumference tended to be lower among non-Hispanic White women overall and lower among cancer-free non-Hispanic Black women compared to non-Hispanic Black women with CRC. We did not observe differences in perceived stress between groups.

**Figure 1:**
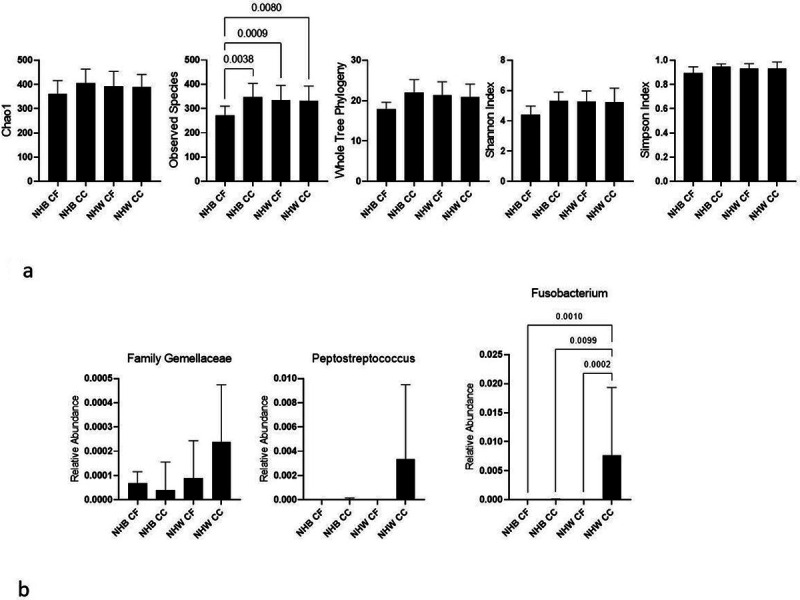
1a. Fecal bacteria alpha diversity metrics by race-cancer status combinations. Chao1, p=0.611; Observed Species, p=0.018; Whole Tree Phylogeny, p=0.012; Shannon Index, p=0.027; Simpson Index, p=0.094 NHB, non-Hispanic Black; CF, Cancer-Free; CC, Colon Cancer; NHW, non-Hispanic White P values not listed are greater than 0.05 1b. Fecal bacteria relative abundance by race-cancer status combinations. NHB, non-Hispanic Black; CF, Cancer-Free; CC, Colon Cancer; NHW, non-Hispanic White P values not listed are greater than 0.05

Associations of alpha diversity with CRC among non-Hispanic Black and non-Hispanic White women combined are presented in Table 2. Alpha diversity was positively associated with CRC, though the findings were generally not statistically significant and the estimates unstable. For example, for every 1-SD increase in the Shannon Index, there was an estimated non-statistically significant 2.1-fold higher odds of CRC (95% CI: 0.68, 8.24). In a comparison of the alpha diversity estimates by race, cancer-free non-Hispanic Black women tended to have the lowest diversity among the groups (Fig. 1A). There were no statistically significant associations of the beta diversity distance matrices (e.g., overall microbiome composition) with CRC (*all P*> 0.50 for Bray-Curtis, Weighted Unifrac, and Unweighted Unifrac distance; data not shown).

**Figure 2:**
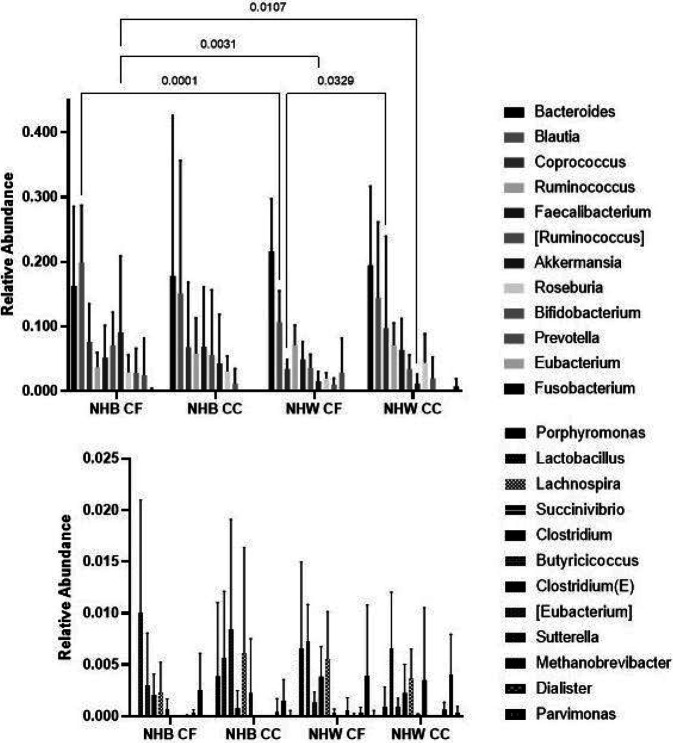
Fecal bacteria genera relative abundance by race-cancer status combinations. NHB, non-Hispanic Black; CF, Cancer-Free; CC, Colon Cancer; NHW, non-Hispanic White P values not listed are greater than 0.05

**Figure 3:**
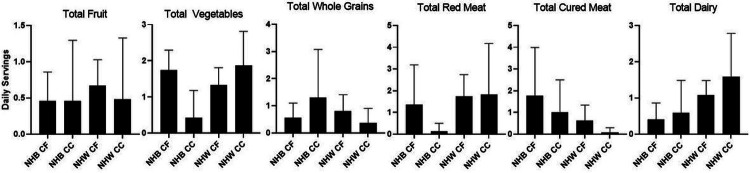
Total daily servings of food groups of women reported by race-cancer status combinations. Total Fruit, p=0.651; Total vegetables, p=0.018; Total whole grains, p=0.446; Total red meat, p=0.457; Total cured meat, p=0.030; Total dairy, p=0.054 NHB, non-Hispanic Black; CF, Cancer-Free; CC, Colon Cancer; NHW, non-Hispanic White P values not listed are greater than 0.05

Associations of the relative abundance of *a priori*-selected taxa with CRC are presented in Table 3. Though no bacteria were statistically significantly associated with CRC after correction for multiple testing, notably, *Fusobacteria*, *Gemellaceae*, and *Peptostreptococcus* were strongly, positively associated with CRC, similar to previous literature (Fig. 1b).^6,36^ Mean relative abundances of individual genera are presented by race-cancer combination in Fig. 2. Non-Hispanic White CRC cases tended to have higher relative abundance of *Fusobacteria, Gemellaceae,* and *Peptostreptococcus* compared to all other race-cancer combination groups. For example, mean relative abundance of *Fusobacterium* abundance was 0.767% among non-Hispanic White women with CRC compared 0.002% among cancer-free non-Hispanic White women (p = 0.0002); whereas, there were no statistically significant differences in *Fusobacterium* abundance among non-Hispanic Black women with and without CRC. As shown in **Supplemental Table 1**, relative abundance of *[Eubacterium]* was higher, on average, among non-Hispanic Black women with CRC compared to non-Hispanic White women with (p = 0.026) and without (p = 0.018) CRC. Total calories and macronutrients, including total sugar and total fiber are reported in Table 4. Although non-Hispanic White women with CRC reported average daily total calories approximately 400 calories more than the other groups, this finding was not statistically significant. No post-hoc differences between groups in any of these nutrients reached statistical significance after Bonferroni correction. Daily intake of food groups relevant to CRC by race-cancer status are presented in Fig. 3. Overall, strong between-group differences were observed for total vegetables and cured meats, while dairy was only marginally different across groups. Post hoc between group differences were only observed in total vegetables, in which non-Hispanic White women with CRC reported an almost four-fold higher consumption compared to non-Hispanic Black women with CRC (p = 0.049).

Finally, we sought to estimate the association of total perceived stress with relative abundance of the genera and alpha diversity controlling for cancer status, age, race, and BMI. Perceived stress was inversely associated with Shannon index, Simpson Index, relative abundance of *Coprococcus, Faecalibacterium, Roseburia, Lachnospira, Butyricicoccus*, and *Sutterella*, and positively associated with *[Ruminococcus] [Eubacterium],* and Parvimonas (Table 5).

## Discussion

The present study reports differences in alpha diversity and CRC-associated signatures of the gut microbiome when comparing Black and White women with a new CRC diagnosis to those who are cancer-free. We observed significantly lower alpha diversity among cancer-free Black women compared to all other race-cancer status combinations. One of the more intriguing findings was that a higher relative abundance of *Fusobacterium*, a genus which tends to be enriched among individuals with CRC,^37^ was only associated with CRC among White women, but not Black women. These findings highlight potentially differences in microbiome-CRC associations, as explained below. Associations between perceived stress and several genera were also observed.

Our findings related to alpha diversity were consistent with much of the limited literature that has specifically compared alpha diversity of generally healthy Black and White women. Other researchers have reported racial differences in alpha diversity including a study by Brooks et al which also reported lower alpha diversity among Black women.^17^ In contrast, a 2018 study conducted by our team found no differences in alpha diversity when comparing Black and White women.^13^ However, the average age of women in the 2018 study was 40 years old whereas women in the present study were in their early 60s, on average. Given that aging is associated with changes in the gut microbiota,^38^ the age difference of study participants may explain some of the observed differences. Our findings may also suggest a potential interaction between race and age in the evolution of the gut microbiota over time, though this requires further investigation. Related to CRC status, studies suggest that lower alpha diversity is observed among individuals with CRC ^6,39^ which is counter to our study finding. Reasons for this are not fully understood, but may suggest that lower alpha diversity among Black women may predispose them to greater risk for developing CRC.

Our assessment of CRC-associated genera yielded interesting findings that might suggest that associations between characteristics of the gut microbiome and CRC may differ by race. For example, *Fusobacterium*, a genus that is commonly enriched among individuals with CRC ^40^, was significantly higher among White women with CRC compared to all other groups, including Black women with CRC. We also observed a higher relative abundance of *Eubacterium* among black women with CRC compared to all other race-cancer status combinations. This finding is consistent with previous studies that found *Eubacterium* to be positively correlated with CRC.^39,41^ It is plausible that exposures associated with race (e.g., diet, stress, racism, environmental exposures) may indeed influence the composition of the gut microbiome. For example, while statistically significant differences in diet measures or stress by race were not observed among our study population, previous research has suggested that Black and White women consume different dietary patterns or display different diet quality^42^ and that Black women have elevated chronic stress compared to White women.^43^ Research also shows that dietary pattern is associated with the gut microbiota.^42,44^ Similarly, stress has been shown to be associated with the composition of the gut microbiota ^45-47^ including in our findings in the current study. Thus, it is plausible that racial differences in these exposures could contribute to differences in associations between the gut microbiome and risk for CRC.

This study was limited by small sample size which may have reduced our ability to detect statistically significant differences. We are also limited, as are the vast majority of current human microbiome and cancer risk studies, by the potential that cancer may have altered the microbiome among cases. Thus, it remains unclear whether observed characteristics of the gut microbiome contributed to the etiology of the cancer or occurred because of the cancer. Lastly, lack of information on medication usage was a study limitation. While antibiotic or probiotic use was assessed and led to the exclusion of participants with recent use, our study did not account for other medications that may affect the gut microbiota. Still, this study has several strengths. First, the racial diversity of the study sample allows for the examination of the gut microbiome as a contributor to health disparities. Next, the stringent matching criteria helped minimize the effect of potential confounding factors although some residual confounding possibly remained. Additionally, we were able to link the microbiome data with other behavioral measures enhancing our ability to explore and understand biobehavioral mechanisms contributing to microbiota-CRC associations.

## Conclusions

This analysis reveals that bacteria previously associated with CRC were generally highest among White women with CRC with the exception of *Eubacterium* which was highest among Black women with CRC. Our findings suggest that previous findings about CRC-signatures in the gut microbiome may be largely driven by racially homogenous groups, i.e., White individuals, and that microbiome-CRC associations may differ by racial group. Additional large, racially diverse population-based studies are needed to determine if previously identified associations between characteristics of the gut microbiome and CRC are generalizable to Black women and other racial, ethnic, and gender groups.

## Figures and Tables

**Table 1:** Characteristics of non-Hispanic black and white female cancer patients and matched cancer-free controls.

	NHB CF (n = 8)	NHB CC (n = 4)	NHW CF (n = 14)	NHW CC (n = 7)	P value
Age (years)	60.6 (6.3)	64 (8.8)	59.1 (8.6)	59.6 (12.4)	0.88
Body Mass Index (kg/m^2^)	37.5 (8.4) ‡	37.2 (12.8)	27.7 (7.2)	30.9 (6.6)	0.02
Waist Circumference (cm)	104.4 (12.3) ‡	111 (14.7)	89.1 (1 5.4) †	110.1 (11.6)	0.006
Perceived Stress Scale (total)	15.4 (10.4)	12.8 (9.6)	15.4 (11)	13.1 (11.1)	0.948

NHB, non-Hispanic Black; CF, Cancer-Free; CC, colorectal cancer; NHW, non-Hispanic White

† within race difference

‡ between race difference

**Table 2:** Associations of alpha diversity estimates with colorectal cancer among black and white women (N = 33; 11 colorectal cancer cases, and 22 cancer-free controls)

Alpha Diversity Metric (range)	Colorectal cancer	Cancer-free	Unadjusted		Adjusted	
	n (%)	n (%)	OR (95% CI)	P-value	OR (95% CI)^b^	P-value
	11	22				
Observed						
Continuous [mean (SD)]	[338.36 (56.55)]	[311.18 (61.50)]	1.00 (1.00, 1.02)	0.23	1.01 (1.00, 1.03)	0.21
Quantile 1 (185–310)	3 (27.3)	11 (50.0)	1.00 (referent)	0.22	1.00 (referent)	0.14
Quantile 2 (312–428)	8 (72.7)	11 (50.0)	2.70 (0.59, 14.74)		4.48 (0.71, 43.49)	
Shannon						
Continuous [mean (SD)]	[5.25 (0.80)]	[4.96 (0.78)]	1.70 (0.65, 5.36)	0.30	2.06 (0.68, 8.24)	0.24
Quantile 1 (3.19–5.06)	3 (27.3)	11 (50.0)	1.00 (referent)	0.22	1.00 (referent)	0.12
Quantile 2 (5.06–6.22)	8 (72.7)	11 (50.0)	2.70 (0.59, 14.74)		4.38 (0.76, 35.56)	
PD Whole Tree						
Continuous [mean (SD)]	[21.36 (3.04)]	20.12 (3.28)	1.10 (0.90, 1.48)	0.29	1.16 (0.90, 1.58)	0.27
Quantile 1 (14.06–19.37)	1 (9.1)	11 (50.0)	1.00 (referent)	0.04	1.00 (referent)	0.03
Quantile 2 (19.38–26.75)	10 (90.9)	11 (50.0)	10.00 (1.52, 199.93)		26.72 (2.34, 1,084)	

Alpha diversity measures were dichotomized based on median value among the matched cancer-free controls.

b Covariates for logistic regression models included: age(continuous), race (black vs. white), and BMI (continuous).

Abbreviations:

CI, confidence interval; OR, odds ratio; PD, Phylogenetic Diversity

**Table 3:** Associations of phylum and genus level relative abundances with colorectal cancer among Black and White women (N = 33; 11 colorectal cancer cases, and 22 cancer-free controls)

	Colorectal cancer	Cancer-free	Unadjusted		Adjusted	
	Mean % relative abundance (SD)	Mean % relative abundance (SD)	OR (95% CI)	P- value	OR (95% CI)^b^	P- value
	11	22				
Phylum						
Firmicutes	66.91 (16.90)	58.80 (14.33)	9.30 (0.47, 353.87)	0.18	10.52 (0.52, 417.32)	0.15
Bacteroidetes	22.97 (14.39)	27.70 (16.59)	0.88 (0.53, 1.49)	0.61	0.88 (0.49 1.56)	0.64
Proteobacteria	4.19 (5.81)	3.76 (6.04)	0.88 (0.53, 1.45)	0.61	0.90 (0.52, 1.52)	0.68
Verrucomicrobia	2.34 (3.27)	4.32 (9.17)	0.97 (0.72, 1.31)	0.84	0.98 (0.71, 1.36)	0.88
Actinobacteria	2.67 (3.33)	3.90 (5.76)	0.94 (0.51, 1.68)	0.82	0.96 (0.53, 1.75)	0.90
Archaea;Euryarchaeota	0.21 (0.34)	0.43 (1.22)	1.10 (0.74, 1.51)	0.78	1.08 (0.75, 1.60)	0.66
Tenericutes	0.03 (0.06)	0.40 (1.29)	0.61 (0.29, 1.02)	0.10	0.56 (0.26, 0.95)	0.06
Cyanobacteria	0.01 (0.05)	0.31 (0.77)	0.88 (0.58, 1.28)	0.53	0.91 (0.59, 1.36)	0.65
Bacteria Other	0.08 (0.09)	0.23 (0.63)	1.20 (0.79, 1.96)	0.39	1.34 (0.81, 2.33)	0.27
Fusobacteria	0.49 (1.05)	0.00 (0.00)	6.00 (1.93 62.75)	0.03	8.16 (2.24, 186.35)	0.03
Phylum; Class:Order;Family;Genus^a^						
Bacteroides	18.86 (13.33)	19.70 (14.15)	0.95 (0.59, 1.59)	0.82	0.96 (0.57, 1.67)	0.88
Blautia	14.64 (12.12)	14.04 (10.06)	1.10 (0.42, 3.14)	0.80	1.19 (0.40, 3.57)	0.75
Coprococcus	8.67 (12.47)	4.98 (4.94)	1.50 (0.71, 3.61)	0.28	1.52 (0.66, 3.85)	0.33
Ruminococcus	6.62 (3.51)	5.89 (4.79)	1.40 (0.77, 3.18)	0.33	1.41 (0.75, 3.34)	0.34
Faecalibacterium	6.59 (5.12)	5.02 (5.07)	1.30 (0.89, 2.29)	0.25	1.32 (0.88, 2.38)	0.26
[Ruminococcus]	4.22 (4.06)	4.88 (4.84)	0.72 (0.28, 1.72)	0.47	0.61 (0.21, 1.63)	0.34
Akkermansia	2.32 (3.26)	4.31 (9.17)	0.98 (0.73, 1.32)	0.89	0.99 (0.72, 1.37)	0.94
Roseburia	3.90 (3.88)	2.34 (2.22)	1.80 (0.95, 4.30)	0.13	1.83 (0.94, 4.66)	0.13
Bifidobacterium	1.68 (2.91)	1.69 (3.04)	1.00 (0.73, 1.45)	0.90	1.06 (0.74, 1.53)	0.76
Porphyromonas	0.22 (0.62)	0.00 (0.00)	1.50 (0.85, 2.95)	0.21	1.68 (0.92, 3.74)	0.12
Prevotella	0.01 (0.01)	2.75 (8.26)	0.73 (0.41, 1.03)	0.15	0.74 (0.41, 1.06)	0.18
Lactobacillus	0.09 (0.21)	0.05 (0.08)	1.10 (0.49, 2.24)	0.83	1.14 (0.48, 2.60)	0.75
Pseudoramibacter_Eubacterium	0.01 (0.02)	0.11 (0.21)	0.79 (0.48, 1.20)	0.30	0.82 (0.49, 1.29)	0.41
Clostridium	0.17 (0.25)	0.32 (0.43)	0.77 (0.47, 1.20)	0.25	0.76 (0.45, 1.23)	0.27
Lachnospira	0.45 (0.45)	0.44 (0.68)	1.10 (0.78, 1.73)	0.56	1.11 (0.77, 1.74)	0.60
Butyricicoccus	0.00 (0.00)	0.01 (0.02)	0.74 (0.27, 1.85)	0.52	0.67 (0.22, 1.81)	0.44
Clostridium	0.06 (0.08)	0.04 (0.07)	1.70 (1.01, 3.45)	0.07	1.75 (1.00, 3.62)	0.08
[Eubacterium]	0.36 (0.53)	0.20 (0.38)	1.10 (0.70, 1.69)	0.72	1.28 (0.76, 2.30)	0.37
Fusobacterium	0.49 (1.05)	0.00 (0.00)	6.00 (1.93, 62.75)	0.03	8.16 (2.24, 186.35)	0.03
Sutterella	0.62 (0.51)	0.83 (0.91)	1.10 (0.80, 1.51)	0.66	1.08 (0.78, 1.56)	0.64
Succinivibrio	0.00 (0.00)	0.04 (0.17)	1.00 (0.38, 2.25)	1.00	1.03 (0.40, 2.38)	0.94
Methanobrevibacter	0.20 (0.33)	0.42 (1.19)	1.10 (0.74, 1.52)	0.78	1.08 (0.75, 1.60)	0.67
Gemellaceae (Family)	0.02 (0.02)	0.01 (0.02)	2.00 (0.99, 4.95)	0.08	2.32 (1.07, 6.20)	0.05
Peptostreptococcus	0.22 (0.54)	0.00 (0.00)	1.80 (1.02, 4.11)	0.08	2.16 (1.14, 5.12)	0.04
Dialister	0.31 (0.35)	0.34 (0.58)	1.10 (0.78, 1.46)	0.70	1.09 (0.78, 1.57)	0.62
Parvimonas	0.03 (0.05)	0.01 (0.04)	1.00 (0.43, 2.47)	0.92	1.10 (0.42, 2.88)	0.84

Relative abundance values were transformed using the centered log-ratio transformation prior to conducting logistic regression to facilitate interpretation

b Covariates for logistic regression models included: age (continuous), race (black vs. white), and BMI (continuous).

Abbreviations: CI, confidence interval; OR, odds ratio

**Table 4:** Total Calories and macronutrients of women with and without colorectal cancer

	NHB CF (n = 8)	NHB CC (n = 4)	NHW CF (n = 14)	NHW CC (n = 7)	P value
Total Calories (kcal)	1617.7 (639.8)	1737.6 (641.3)	1695.1 (477)	2103.5 (928.8)	0.761
Total Protein (grams)	64.4 (25.3)	57.5 (24.5)	69.1 (18.2)	76.2(31)	0.709
Total Fat (grams)	66.5 (35.6)	65.6 (24.7)	64.8 (22.4)	102.2 (40.7)	0.155
Total Carbohydrate (grams)	194.4 (67.6)	235.6 (94.5)	197.4 (66.4)	224.7 (121.2)	0.841
Total Sugar (grams)	88 (51.4)	101.9 (47.4)	94.3 (46.5)	109.3 (65)	0.865
Total Fiber (grams)	16.1 (6.9)	20.1 (15.5)	13.8 (5.6)	1 5.3 (6.6)	0.841

NHB, non-Hispanic Black; CF, Cancer-Free; CC, Colorectal Cancer; NHW, non-Hispanic White

**Table 5:** Partial Spearman Correlations between Total Perceived Stress Scale Score, alpha diversity, and relative abundance of target ASVs

	Rho, p value
Alpha Diversity Metric	
chao1	−0.137, p = 0.405
Observed_species	−0.375, p = 0.019
PD_whole_tree	−0.304, p = 0.060
shannon	−0.422, p = 0.007
simpson	−0.444, p = 0.005
Phylum; Class:Order;Family;Genus^a^	
Bacteroidetes;Bacteroidia;Bacteroidales;Bacteroidaceae;Bacteroides	−0.137, p = 0.406
Firmicutes;Clostridia;Clostridiales;Lachnospiraceae;Blautia	0.122, p = 0.461
Firmicutes;Clostridia;Clostridiales;Lachnospiraceae;Coprococcus	−0.449, p = 0.004
Firmicutes;Clostridia;Clostridiales;Ruminococcaceae;Ruminococcus	−0.159, p = 0.333
Firmicutes;Clostridia;Clostridiales;Ruminococcaceae;Faecalibacterium	−0.634, p < 0.001
Firmicutes;Clostridia;Clostridiales;Lachnospiraceae;[Ruminococcus]	0.751, p < 0.001
Verrucomicrobia;Verrucomicrobiae;Verrucomicrobiales;Verrucomicrobiaceae;Akkermansia	−0.139, p = 0.399
Firmicutes;Clostridia;Clostridiales;Lachnospiraceae;Roseburia	−0.573, p < 0.001
ActinoActinoBifidobacteriales;Bifidobacteriaceae;Bifidobacterium	0.247, p = 0.129
Bacteroidetes;Bacteroidia;Bacteroidales;Porphyromonadaceae;Porphyromonas	0.055, p = 0.74
Bacteroidetes;Bacteroidia;Bacteroidales;Prevotellaceae;Prevotella	0.119, p = 0.471
Firmicutes;Bacilli;Lactobacillales;Lactobacillaceae;Lactobacillus	−0.071, p = 0.665
Firmicutes;Clostridia;Clostridiales;Eubacteriaceae;Pseudoramibacter_Eubacterium	−0.172, p = 0.296
Firmicutes;Clostridia;Clostridiales;Lachnospiraceae;Clostridium	0.161, p = 0.327
Firmicutes;Clostridia;Clostridiales;Lachnospiraceae;Lachnospira	−0.461, p = 0.003
Firmicutes;Clostridia;Clostridiales;Ruminococcaceae;Butyricicoccus	−0.394, p = 0.013
Firmicutes;Erysipelotrichi;Erysipelotrichales;Erysipelotrichaceae;Clostridium	0.185, p = 0.26
Firmicutes;Erysipelotrichi;Erysipelotrichales;Erysipelotrichaceae;[Eubacterium]	0.604, p < 0.001
FusoFusobacteriia;Fusobacteriales;Fusobacteriaceae;Fusobacterium	−0.133, p = 0.421
ProteoBetaproteoBurkholderiales;Alcaligenaceae;Sutterella	−0.605, p < 0.001
ProteoGammaproteoAeromonadales;Succinivibrionaceae;Succinivibrio	−0.188, p = 0.252
Archaea;Euryarchaeota;MethanoMethanobacteriales;Methanobacteriaceae;Methanobrevibacter	−0.143, p = 0.386
Firmicutes;Bacilli;Gemellales;Gemellaceae;NA	0.104, p = 0.529
Firmicutes;Clostridia;Clostridiales;Peptostreptococcaceae;Peptostreptococcus	−0.136, p = 0.408
Firmicutes;Clostridia;Clostridiales;Veillonellaceae;Dialister	−0.047, p = 0.776
Firmicutes;Clostridia;Clostridiales;[Tissierellaceae];Parvimonas	0.42, p = 0.008

## Data Availability

All data and codes will be made available upon request.

## References

[1] Cronin KA, Scott S, Firth AU, Annual report to the nation on the status of cancer, part 1: National cancer statistics. Cancer. Oct 27 2022;doi:10.1002/cncr.34479 PMC10092838 36301149

[2] Augustus GJ, Ellis NA. Colorectal Cancer Disparity in African Americans: Risk Factors and Carcinogenic Mechanisms. Am J Pathol. Feb 2018;188(2):291–303. doi:10.1016/j.ajpath.2017.07.023 29128568 PMC5785537

[3] Ollberding NJ, Nomura AM, Wilkens LR, Henderson BE, Kolonel LN. Racial/ethnic differences in colorectal cancer risk: the multiethnic cohort study. Int J Cancer. Oct 15 2011;129(8):1899–906. doi:10.1002/ijc.25822 21128280 PMC3126877

[4] Centers for Disease Control and Prevention. What are the risk factors for colorectal cancer? Updated February 17, 2022. Accessed November 18, 2022. https://www.cdc.gov/cancer/colorectal/basic_info/risk_factors.htm

[5] Cipe G, Idiz UO, Firat D, Bektasoglu H. Relationship between intestinal microbiota and colorectal cancer. World journal of gastrointestinal oncology. Oct 15 2015;7(10):233–40. doi:10.4251/wjgo.v7.i10.233 26483877 PMC4606177

[6] Ahn J, Sinha R, Pei Z, Human gut microbiome and risk for colorectal cancer. Journal of the National Cancer Institute. Dec 18 2013;105(24):1907–11. doi:10.1093/jnci/djt300 24316595 PMC3866154

[7] Song M, Chan AT, Sun J. Influence of the Gut Microbiome, Diet, and Environment on Risk of Colorectal Cancer. Gastroenterology. Jan 2020;158(2):322–340. doi:10.1053/j.gastro.2019.06.048 31586566 PMC6957737

[8] Zisman TL, Rubin DT. Colorectal cancer and dysplasia in inflammatory bowel disease. World journal of gastroenterology : WJG. May 7 2008;14(17):2662–9. doi:10.3748/wjg.14.2662 18461651 PMC2709054

[9] Brennan CA, Garrett WS. Gut Microbiota, Inflammation, and Colorectal Cancer. Annu Rev Microbiol. Sep 8 2016,70:395–411. doi:10.1146/annurev-micro-102215-095513 27607555 PMC5541233

[10] Montoya-Williams D, Lemas DJ, Spiryda L, The Neonatal Microbiome and Its Partial Role in Mediating the Association between Birth by Cesarean Section and Adverse Pediatric Outcomes. Neonatology. May 22 2018;114(2):103–111. doi:10.1159/000487102 29788027 PMC6532636

[11] Rutayisire E, Huang K, Liu Y, Tao F. The mode of delivery affects the diversity and colonization pattern of the gut microbiota during the first year of infants' life: a systematic review. BMC gastroenterology. Jul 30 2016;16(1):86. doi:10.1186/s12876-016-0498-0 27475754 PMC4967522

[12] Singh RK, Chang HW, Yan D, Influence of diet on the gut microbiome and implications for human health. Journal of translational medicine. Apr 8 2017;15(1 ):73. doi:10.1186/s12967-017-1175-y 28388917 PMC5385025

[13] Carson TL, Wang F, Cui X, Associations between Race, Perceived Psychological Stress, and the Gut Microbiota in a Sample of Generally Healthy Black and White Women: A Pilot Study on the Role of Race and Perceived Psychological Stress. Psychosomatic medicine. Jun 12 2018;doi:10.1097/psy.0000000000000614 PMC6113071 29901485

[14] Clauss M, Gérard P, Mosca A, Leclerc M. Interplay Between Exercise and Gut Microbiome in the Context of Human Health and Performance. Front Nutr. 2021;8:637010. doi:10.3389/fnut.2021.637010 34179053 PMC8222532

[15] Weersma RK, Zhernakova A, Fu J. Interaction between drugs and the gut microbiome. Gut. Aug 2020;69(8):1510–1519. doi:10.1136/gutjnl-2019-320204 32409589 PMC7398478

[16] Deschasaux M, Bouter KE, Prodan A, Depicting the composition of gut microbiota in a population with varied ethnic origins but shared geography. Nature medicine. Oct 2018;24(10):1526–1531. doi:10.1038/S41591-018-0160-1 30150717

[17] Brooks AW, Priya S, Blekhman R, Bordenstein SR. Gut microbiota diversity across ethnicities in the United States. PLoS Biol. Dec 2018;16(12):e2006842. doi:10.1371/journal.pbio.2006842 30513082 PMC6279019

[18] Sánchez-Alcoholado L, Ramos-Molina B, Otero A, The Role of the Gut Microbiome in Colorectal Cancer Development and Therapy Response. Cancers (Basel). May 29 2020;12(6)doi:10.3390/cancers12061406 PMC7352899 32486066

[19] Costantini L, Molinari R, Farinon B, Merendino N. Impact of Omega-3 Fatty Acids on the Gut Microbiota. Int J Mol Sci. Dec 7 2017;18(12)doi:10.3390/ijms18122645 PMC5751248 29215589

[20] Borrello K, Lim U, Park SY, Dietary Intake Mediates Ethnic Differences in Gut Microbial Composition. Nutrients. Feb 4 2022;14(3)doi:10.3390/nu14030660 PMC8840192 35277019

[21] Byrd DA, Carson TL, Williams F, Vogtmann E. Elucidating the role of the gastrointestinal microbiota in racial and ethnic health disparities. Genome biology. Aug 3 2020;21(1):192. doi:10.1186/s13059-020-02117-w 32746938 PMC7397581

[22] Bennett G, Bardon LA, Gibney ER. A Comparison of Dietary Patterns and Factors Influencing Food Choice among Ethnic Groups Living in One Locality: A Systematic Review. Nutrients. Feb 23 2022;14(5)doi:10.3390/nu14050941 PMC8912306 35267916

[23] Newby PK, Noel SE, Grant R, Judd S, Shikany JM, Ard J. Race and region have independent and synergistic effects on dietary intakes in black and white women. Nutrition journal. Apr 13 2012;11:25. doi:10.1186/1475-2891-11-25 22500645 PMC3386860

[24] Veettil SK, Wong TY, Loo YS, Role of Diet in Colorectal Cancer Incidence: Umbrella Review of Meta-analyses of Prospective Observational Studies. JAMA Netw Open. Feb 1 2021;4(2):e2037341. doi:10.1001/jamanetworkopen.2020.37341 33591366 PMC7887658

[25] American Psychological Association APA Working Group on Stress and Health Disparities (2017). Stress and health disparities: Contexts, mechanisms, and interventions among racial/ethnic and low-socioeconomic status populations. Retrieved from http://www.apa.org/pi/health-disparities/resources/stress-report.aspx. http://www.apa.org/pi/health-disparities/resources/stress-report.aspx

[26] Cohen S, Kamarck T, Mermelstein R. A global measure of perceived stress. Journal of Health and Social Behavior. 1983;24(4):385–396. 6668417

[27] Cohen S, Williamson G. Perceived Stress in a Probability Sample of the United States. In: Spacapan S, Oskamp S, eds. The Social Psychology of Health. Sage; 1988.

[28] Subar AF, Kirkpatrick SI, Mittl B, The Automated Self-Administered 24-hour dietary recall (ASA24): a resource for researchers, clinicians, and educators from the National Cancer Institute. Journal of the Academy of Nutrition and Dietetics. Aug 2012;112(8):1134–7. doi:10.1016/j.jand.2012.04.016 22704899 PMC3721511

[29] Callahan BJ, McMurdie PJ, Rosen MJ, Han AW, Johnson AJ, Holmes SP. DADA2: High-resolution sample inference from Illumina amplicon data. Nature methods. Jul 2016;13(7):581–3. doi:10.1038/nmeth.3869 27214047 PMC4927377

[30] Schloss PD, Westcott SL, Ryabin T, Introducing mothur: open-source, platform-independent, community-supported software for describing and comparing microbial communities. Research Support, Non-U S Gov't. Applied and environmental microbiology. 2009;75(23):7537–41. 19801464 10.1128/AEM.01541-09 PMC2786419

[31] Wang Q, Garrity GM, Tiedje JM, Cole JR. Naive Bayesian classifier for rapid assignment of rRNA sequences into the new bacterial taxonomy. Applied and environmental microbiology. Aug 2007,73(16):5261–7. doi:10.1128/aem.00062-07 17586664 PMC1950982

[32] Cole JR, Wang Q, Cardenas E, The Ribosomal Database Project: improved alignments and new tools for rRNA analysis. Nucleic acids research. Jan 2009;37(Database issue):D141–5. doi:10.1093/nar/gkn879 19004872 PMC2686447

[33] Yilmaz R Parfrey LW, Yarza P, The SILVA and "All-species Living Tree Project (LTP)" taxonomic frameworks. Nucleic acids research. Jan 2014;42(Database issue):D643–8. doi:10.1093/nar/gkt1209 24293649 PMC3965112

[34] Kumar R, Eipers R Little RB, Getting started with microbiome analysis: sample acquisition to bioinformatics. Current protocols in human genetics. Jul 14 2014;82:18.8.1–29. doi:10.1002/0471142905.hg1808s82 PMC4383038 25042718

[35] Bolyen E, Rideout JR, Dillon MR, Reproducible, interactive, scalable and extensible microbiome data science using QIIME 2. NatBiotechnol. Aug 2019;37(8):852–857. doi:10.1038/s41587-019-0209-9 PMC7015180 31341288

[36] Wirbel J, Pyl PT, Kartal E, Meta-analysis of fecal metagenomes reveals global microbial signatures that are specific for colorectal cancer. Nature medicine. Apr 2019;25(4):679–689. doi:10.1038/s41591-019-0406-6 PMC7984229 30936547

[37] Yang Z, Ji G. Fusobacterium nucleatum-positive colorectal cancer. Oncol Lett. Aug 2019;18(2):975–982. doi:10.3892/ol.2019.10433 31423156 PMC6607099

[38] Yatsunenko T, Rey FE, Manary MJ, Human gut microbiome viewed across age and geography. Nature. May 9 2012;486(7402):222–7. doi:10.1038/nature11053 22699611 PMC3376388

[39] Ai D, Pan H, Li X, Gao Y, Liu G, Xia LC. Identifying Gut Microbiota Associated With Colorectal Cancer Using a Zero-Inflated Lognormal Model. Frontiers in microbiology. 2019;10:826. doi:10.3389/fmicb.2019.00826 31068913 PMC6491826

[40] Bashir A, Miskeen AY, Bhat A, Fazili KM, Ganai BA. Fusobacterium nucleatum: an emerging bug in colorectal tumorigenesis. European journal of cancer prevention : the official journal of the European Cancer Prevention Organisation (ECP). Sep 2015;24(5):373–85. doi:10.1097/cej.0000000000000116 25569450

[41] Wang Y, Wan X, Wu X, Zhang C, Liu J, Hou S. Eubacterium rectale contributes to colorectal cancer initiation via promoting colitis. Gut pathogens. Jan 12 2021;13(1 ):2. doi:10.1186/s13099-020-00396-z 33436075 PMC7805161

[42] Little RB, Murillo AL, Van Der Pol WJ, Diet Quality and the Gut Microbiota in Women Living in Alabama. American journal of preventive medicine. Jul 2022;63(1 Suppl 1):S37–s46. doi:10.1016/j.amepre.2022.02.015 35725139 PMC9219556

[43] American Psychological Association. Stress and Gender. 2014. Stress in America. http://www.apa.org/news/press/releases/stress/2010/gender-stress.pdf

[44] Bolte LA, Vich Vila A, Imhann F, Long-term dietary patterns are associated with pro-inflammatory and anti-inflammatory features of the gut microbiome. Gut. Jul 2021 ;70(7):1287–1298. doi:10.1136/gutjnl-2020-322670 33811041 PMC8223641

[45] Carson TL, Wang F, Cui X, Racial comparisons of the gut microbiota of generally healthy black and white women for insights into colorectal cancer disparities 2017:

[46] Madison A, Kiecolt-Glaser JK. Stress, depression, diet, and the gut microbiota: human-bacteria interactions at the core of psychoneuroimmunology and nutrition. Curr Opin Behav Sci. Aug 2019;28:105–110. doi:10.1016/j.cobeha.2019.01.011 32395568 PMC7213601

[47] Geng S, Yang L, Cheng F, Gut Microbiota Are Associated With Psychological Stress-Induced Defections in Intestinal and Blood-Brain Barriers. Frontiers in microbiology. 2019;10:3067. doi:10.3389/fmicb.2019.03067 32010111 PMC6974438

